# yGPS-P: A Yeast-Based Peptidome Screen for Studying Quality Control-Associated Proteolysis

**DOI:** 10.3390/biom13060987

**Published:** 2023-06-14

**Authors:** Bayan Mashahreh, Shir Armony, Tommer Ravid

**Affiliations:** Department of Biological Chemistry, The Alexander Silberman Institute of Life Sciences, The Hebrew University of Jerusalem, Jerusalem 91904, Israel

**Keywords:** protein quality control, quality control-associated proteolysis, ubiquitin-proteasome system (UPS), degronome, fluorescence-activated cell sorting (FACS), next generation sequencing (NGS)

## Abstract

Quality control-associated proteolysis (QCAP) is a fundamental mechanism that maintains cellular homeostasis by eliminating improperly folded proteins. In QCAP, the exposure of normally hidden *cis*-acting protein sequences, termed degrons, triggers misfolded protein ubiquitination, resulting in their elimination by the proteasome. To identify the landscape of QCAP degrons and learn about their unique features we have developed an unbiased screening method in yeast, termed yGPS-P, which facilitates the determination of thousands of proteome-derived sequences that enhance proteolysis. Here we describe the fundamental features of the yGPS-P method and provide a detailed protocol for its use as a tool for degron search. This includes the cloning of a synthetic peptidome library in a fluorescence-based reporter system, and data acquisition, which entails the combination of Fluorescence-Activated Cell Sorting (FACS) and Next-Generation Sequencing (NGS). We also provide guidelines for data extraction and analysis and for the application of a machine-learning algorithm that established the evolutionarily conserved amino acid preferences and secondary structure propensities of QCAP degrons.

## 1. Introduction

Protein misfolding and aggregation are fundamental problems that every cell faces. Consequently, cells employ various co- and post-translational protein quality control (PQC) mechanisms to mitigate this problem. These include molecular chaperones that can repair misfolded proteins and return them to a functional state and proteolytic systems that recognize misfolded proteins and eliminate them through degradation. PQC pathways have evolved to be extraordinarily robust because they must maintain protein homeostasis (proteostasis) indefinitely, despite stochastic external and internal variations that challenge protein folding. When PQC pathways demonstrate low performance or when they are otherwise overwhelmed, misfolded proteins accumulate and often form insoluble aggregates that can lead to cellular dysfunction [1]. The deleterious consequences of a cell’s failure to cope with misfolded proteins are apparent in all living organisms, from bacteria to humans [2]. The importance of this failure is best highlighted by the >50 devastating human diseases causally linked to protein aggregation [3].

One strategic way eukaryotic cells cope with misfolded proteins burden is by destroying them through Quality Control-Associated Proteolysis (QCAP). For cellular QCAP to function efficiently, cells must have the capacity to destroy misfolded proteins quickly and at the same time ignore normally folded proteins [2]. QCAP is typically executed by the ubiquitin-proteasome system (UPS), which involves specific ubiquitin ligases that recognize and mediate the ubiquitination of misfolded proteins for subsequent destruction by the 26S proteasome [4]. However, the UPS is also involved in the degradation of normally folded proteins for physiological control [5]. This means that, for the UPS to respond correctly to varied physiological conditions, it requires diverse and sometimes conflicting features from its substrates [6]. On the one hand, there is a need for stringent sequence specificity to degrade proteins involved in controlled cellular processes (e.g., transcription, signaling, cell-cycle progression, etc.) with precise temporal and spatial accuracy. On the other hand, any protein can misfold and broad flexibility in the recognition process is essential for rapidly and efficiently targeting proteins undergoing non-native conformational changes for degradation without consequences to normal proteins. Thus, high specificity is still required for QCAP to avert the destruction of normal and fully functional proteins.

A key question is how QCAP pathways handle such a diverse array of misfolded substrates without sacrificing normal proteins. The main hypothesis in the PQC field is that QCAP systems recognize the biophysical phenomenon of exposed hydrophobicity [7]. This is based on the biochemical property that soluble folded proteins place hydrophobic residues in their interior to form a hydrophobic core, away from the aqueous solution. Thus, it is not surprising that the exposure of hydrophobic residues is a key feature of QCAP degrons [8,9,10,11,12]. However, “exposed hydrophobicity” is an exceptionally broad term that can encompass many distinct features that might be overlooked if using this superficial term as a “catch-all” for misfolded protein recognition. In fact, it is our working hypothesis that not all exposed hydrophobicity is the same and that the majority of the eukaryotic proteome contains cryptic QCAP degrons that are exposed upon stress induction.

The main obstacle in identifying QCAP degrons, let alone their mechanism of function, is that they cannot be deduced from basic features of already known degrons, due to their sequence and structural heterogeneity. To overcome these challenges, we have recently implemented the Global Protein Stability peptidome (GPS-P) technology in yeast, termed yGPS-P, to identify QCAP degrons and their cognate degradation machinery [13,14]. In yGPS-P, a synthetic peptide library that emerged from the yeast ORFome is fused C-terminally to a GFP reporter, while a Cherry fluorescent protein precedes the GFP on the same mRNA transcript. While both Cherry and GFP are transcribed to a single mRNA, they are translated separately. Hence, alterations in the GFP/Cherry ratio upon peptide appendage indicate an increased elimination rate of the GFP. We used the yGPS-P system to identify a large cohort of internal degrons and thus to decipher rules governing the stability of the eukaryotic proteome. Here, we provide a detailed protocol of this system from the library design to the revelation of principal features of internal yeast QCAP degrons, including the preferred amino acid and secondary structure properties. 

## 2. Experimental Design

Below described are our considerations for the establishment of the yGPS-P method, which includes three primary steps: (1) Generation of a peptidome library; (2) Data collection (3) Data analysis:

*(1)*  *Generation of a peptidome library*

**Figure 1:**
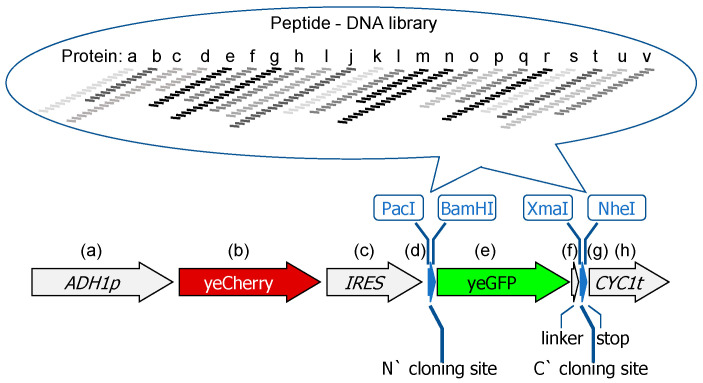
Schematic presentation of yeast GPS-peptidome (yGPS-P). yGPS-P is composed of (**a**) *ADH1* promoter, (**b**) yeast-enhanced Cherry (yeCherry), (**c**) Internal Ribosomal Entry Site (IRES), (**d**) *Pac*I /*Bam*HI N-terminal cloning site, (**e**) yeast-enhanced GFP (yeGFP), (**f**) a pentameric linker (GSAGS), (**g**) *Xma*I/*Nhe*I C-terminal cloning site, (**h**) *CYC1* terminator sequence. A DNA library can be inserted at either the N- or C- termini of yeGFP. Each letter represents different yeast proteins, divided into tiled peptides.

Adaptation of GPS-P to yeast was achieved by us by utilizing a bicistronic gene expression vector, where yeast-enhanced Cherry and similarly, yeast-enhanced GFP are expressed from a single transcript but translated separately [15]. It was possible due to the presence of the 5′ leader of the mRNA-encoding initiation factor eIF4G upstream to yeG [15]. This factor functions as an Internal Ribosome Entry Site (IRES) [16] that enables the initiation of translation in a cap-independent manner (Figure 1). As a source for a peptide library, we employed a set of 326 yeast proteins from various cell compartments. These proteins were chosen since they are part of a protein complex. Initially we hypothesized that these proteins would be enriched with degrons at the protein-protein interaction interface, however, no significant differences in degron abundance were found between monomeric proteins and proteins in a complex (not shown). A list of the selected proteins has been provided as Supplemental Data 1 in a manuscript published by Mashahreh et al. in 2022 [14]. Sequences of the selected proteins were organized as tiled peptides of 17 amino acids (Figure 1). This length was chosen since it is a minimal length that is still anticipated to gain a 2D structure. DNA fragments corresponding to the said peptides were synthesized as an Oligo mixture and inserted into yGPS-P vector at the C-terminus of the stop-codon-less GFP by the Gibson assembly reaction. Library validation and degron presence were previously described [14].

*(2)*  *Data collection*

**Figure 2:**
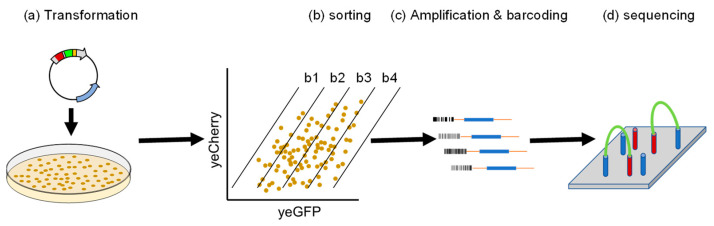
Data collection entails the following steps: (**a**) High-efficiency transformation of yGPS-P into yeast. (**b**) Fluorescence-activated cell sorting into four bins. (**c**) PCR amplification and barcoding of each condition. (**d**) Next-generation sequencing.

Plasmids were transformed into the baker’s yeast *Saccharomyces Cerevisiae*, giving rise to a library of 21,200 different peptides, which is ~90% of the DNA synthesized. Next, the ratio between GFP and Cherry was determined by flow cytometry and/or Fluorescence-Activated Cell Sorting (FACS) (Figure 2a,b). As both Cherry and GFP are expressed from a single transcript, yet translated independently, Cherry levels reflect the basal expression of the reporters while the GFP-to-Cherry ratio determines the relative GFP level that is governed by the fused peptide. Arbitrarily, four distinct populations (Bins 1–4) were defined, exhibiting low to high GFP-to-Cherry ratios, respectively (Figure 2b). Each cell population was isolated by FACS, and purified plasmids were then amplified, barcoded (Figure 2c), and subjected to next-generation sequencing (NGS) (Figure 2d) to identify the DNA content of the corresponding peptides (sequence and abundance) within each bin.

*(3)*  *Data analysis*

**Figure 3:**
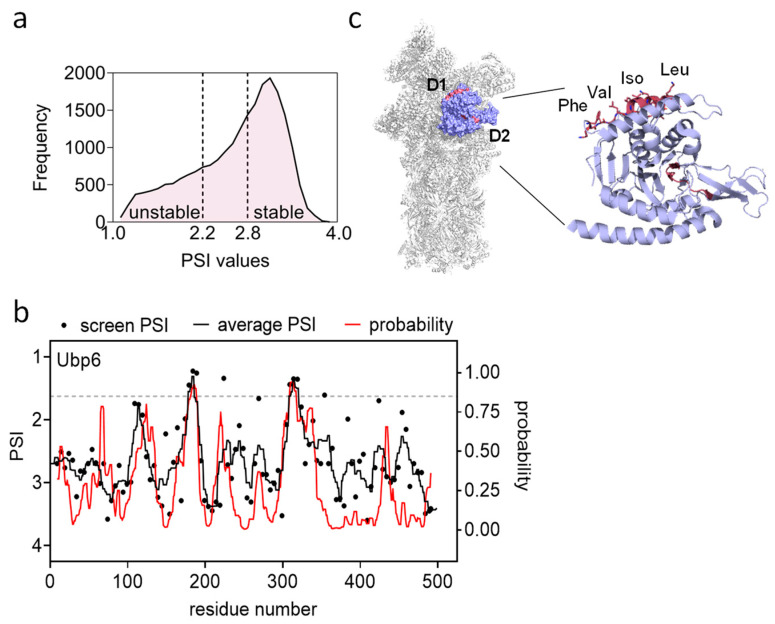
Degron assignment using QCDPred. (**a**) A trendline of peptide densities (number of appearances of PSI scores in clusters of 0.1 units) was plotted against PSI values, and unstable and stable peptides were used as input for machine learning to predict degron probability. (**b**) Analysis of Ubp6 as an example of machine learning outcomes. Two possible degrons were identified by both the experimental and calculated data (cutoff > 0.85 (dashed line)). (**c**) Assignment of predicted degrons into Ubp6 3D structure on the proteasome (PDB 7QO5). Labeled are surface exposed hydrophobic aa on predicted degron I (D1).

NGS data enables the quantification of peptide levels in each FACS bin. With adequate coverage, scoring values were assigned to each peptide, representing their average bin location, termed Protein Stability Index (PSI) [17]. Thus, the PSI value of each peptide is a relative measure of propensity to contribute to the degradation of the otherwise stable GFP. PSI values facilitated the binary classification of each peptide as a degron or non-degron (PSI ≤ 2.2, PSI ≥ 2.8, respectively) (Figure 3a). The resulting classified data was used to train a machine learning model, which provided a predictor for each amino acid within a given protein to be part of a degron (QCDPred [13]). For example, both experimental and QCDPred data for the deubiquitinase (DUB) protein Ubp6, denoted the presence of two potential degrons (Figure 3b, *p* > 0.85). Subsequent assignment of these regions to the Ubp6 3D structure (PDB 7QO5) [18], indicated that one is buried within the protein core while the other forms a surface-exposed hydrophobic stretch that likely interacts with other, yet-to-be-identified, protein(s) (Figure 3c).

## 3. Materials and Equipment

Here we describe all materials and key equipment needed to perform the yGPS-P screen. This includes bacteria and yeast strains (Table 1), Primers (Table 2), Commercial kits (Table 3), chemicals (Table 4), Key equipment (Table 5), and yeast and bacteria growth media (Table 6, Table 7 and Table 8).

Growth media:

**Table 6:** Luria Broth (LB) *.

Reagent	Amount
Trypton	10 gr
Yeast extract	5 gr
NaCl	10 gr
NaOH (10 N)	60 μL

Mix with 1 L of deionized water in a 2 L Erlenmeyer flask and autoclave. Add 100 μg/mL ampicillin when needed.

**Table 7:** Yeast extract Peptone Dextrose (YPD) *.

Reagent	Amount
Peptone	20 gr
Yeast extract	10 gr
Glucose	20 gr
Adenine (0.5%)	4 mL

Mix with 1 L of deionized water in a 2 L Erlenmeyer flask and autoclave.

**Table 8:** Synthetic Defined media without leucine (SD-Leu) *.

Reagent	Amount
Yeast Nitrogen Base without amino acids	1.7 gr
Ammonium Sulfate	5 gr
Glucose	20 gr
Amino acid mix without Leu × 100	10 mL
1% Uracil	4 mL
Adenine (0.5%)	4 mL

Mix with 1 L of deionized water in a 2 L Erlenmeyer flask and autoclave. * Add 2% agar when preparing medium for Petri dishes.

## 4. Detailed Procedure

### 4.1. Library Design and Ordering

As a source for the peptide library, we chose 326 yeast proteins. The coding DNA sequence of each protein was divided into 51 bp fragments with 36 bp overlaps between neighboring oligonucleotides. All fragments were synthesized by LC Sciences (Houston, TX, USA) to be flanked with two primer-binding regions complementary to the yGPS-P expression vector to enable Gibson assembly

### 4.2. Library Amplification

Timing: 3 h.

This step describes the amplification and clean-up of the library pool.

1. Conduct the following PCR to amplify the library:

**Reagent**

**Amount**

**Final Concentration**
x 5 Phusion HF buffer10 µLx 1library pool1 µL25 ngdNTPs1 µL200 µMP1-For2.5 µL500 nMP2-Rev2.5 µL500 nMPhusion polymerase0.5 µL1.0 unitDDW32.5up to 50 µL

2. Run the PCR:

**Steps**

**Temperature**

**Time**

**Cycles**
Initial Denaturation98 °C30 s1Denaturation98 °C10 s18 cyclesAnnealing58 °C30 s
Extension72 °C15 s
Final extension72 °C5 min
Hold4 °Cforever

3. Load 2 μL of the reaction on a 3% TAE/agarose gel and run at constant 100 V for 30 min.

Troubleshooting: if no band is visible, increase the concentration of the library pool or add more cycles (up to 24 cycles total).

4. Clean up the PCR product with NucleoSpin Gel and PCR Clean-up kit, according to the manufacturer’s instructions, and measure the concentration by NanoDrop.

Pause Point: The DNA may be stored at −20 °C.

### 4.3. Vector Linearization

Timing: 1 day

This step describes how to perform the restriction digestion and the clean-up of the plasmid.

5. Set up the restriction digest reaction as follows:

**Reagent**

**Amount**
10X CutSmart buffer8 µL*Nhe*I4 µL*Xma*I4 µLPlasmid5 µgDDWUp to 80 µL

6. Incubate the reaction for 150 min at 37 °C.
7. Mix the reaction with 16 μL of 6× DNA loading dye, load on 1% TAE/agarose gel, and run the gel at 80 V for about 90 min.

Note: Run ~300 ng of undigested plasmid on the agarose gel to check if the plasmid is fully digested. 

Troubleshooting: if the plasmid is not fully digested, increase the restriction duration, and/or add more restriction enzymes.

8. Excise the band of the linearized plasmid from the gel using a sterile scalpel.

*Note*: Perform the cutting as quickly as possible to avoid DNA damage by UV.

9. Extract the plasmids from the gel using the NucleoSpin Gel and PCR Clean-up kit, according to the manufacturer’s protocol. Quantify the product by A260 using a nanodrop spectrophotometer. Expected DNA recovery is approximately 60%.

Pause Point: The DNA may be stored at −20 °C.

### 4.4. Gibson Assembly Reaction and Efficiency Transformation to E. coli

Timing: 3 h.

This step describes the cloning of the library pool into the plasmid by Gibson assembly, followed by clean-up and the transformation into electrocompetent bacteria cells.

10. Prepare the following Gibson reaction:

**Reagent**

**Amount**
x 2 master mix10 µLdigested vector~300 ngAmplified library~20 ngDDWUp to 20 µL

Note: The mole ratio between the vector and amplified library is 1:5. Perform another reaction without the amplified library as a control for the Gibson reaction.

11. Incubate the Gibson assembly reactions for 1 h at 50 °C.
12. Clean the plasmids with AMPure XP beads, according to the manufacturer’s protocol. Elute with ~15 µL of DDW. The expected concentration is ~20 ng/µL.
13. Thaw an ampule of 10-Beta electrocompetent Cells on ice.

Note**:** The electrocompetent cells are very sensitive so do not vortex.

14. Place two electroporation cuvettes (2 mm gap) on ice.
15. Divide 1000 μL of SOC medium into Eppendorf tubes and keep at 42 °C.
16. Add 2 μL of the Gibson product to the pre-chilled cuvettes.
17. Add 20 μL of bacteria to the cuvette.

Note: Do not pipette since it could generate air bubbles and warm the cells.

18. Electroporate the cells with the ECM 399 electroporator system (BTX) using a 2 kV pulse.
19. Immediately, add the recovery medium to the cuvette and invert three times.
20. Transfer the mixture to Eppendorf tubes and incubate at a 37 °C rotating incubator for a 1 h recovery.
21. Spin down the cells and discard 700 μL from the supernatant. Spread the remaining bacteria on 120 × 120 mm Petri dishes containing LB medium with 50 µg/mL ampicillin for plasmid selection.
22. Incubate the plates upside-down for 16 h at 37 °C.

Note: plate the bacteria instead of growing them in a liquid medium to prevent bacteria competition for resources which may lead to the depletion of some sequences.

### 4.5. Purification of Plasmid Library

Timing: 1 day
  23. Add 3 mL LB-medium with ampicillin to each culture plate and scrape the bacteria off all plates into a sterile 50 mL tube.
  24. Freeze glycerol stocks by mixing 10% of the bacteria with 25% sterile glycerol. Put directly to −80 °C.
  25. Centrifuge the remaining cells at 5000× *g* for 15 min at 4 °C.
  26. Perform DNA maxi prep by PureLink^®^ HiPure plasmid filter Midiprep kit according to the manufacturer’s instructions.
  27. Quantify the plasmid DNA using NanoDrop. This yields about 0.6 mg.

Pause Point: The DNA may be stored at −20 °C.

### 4.6. Introducing the Plasmid Library to Yeast Cells

Timing: 1 week
  28. Inoculate a single colony into 5 mL of YPD medium and grow at 30 °C for ~16.
  29. Dilute the culture to 0.2 OD600 in a total volume of 250 mL YPD in an Erlenmeyer flask and allow cells to reach ∼0.7–0.9 OD600.
  30. Pellet cells at 3000× *g* for 5 min at room temperature.
  31. Pour off the supernatant and resuspend the pellet directly in 5 mL of 0.1 M LiAc (with 1 M sorbitol).
  32. Spin cells at 3000× *g* for 5 min at room temperature and discard the supernatant.
  33. Re-suspend the pellet with 2 mL of 0.1 M LiAc (with 1 M sorbitol). Mix 100 μL of yeast-competent cells, 50 μg salmon sperm DNA, and 1 μg of plasmid library in Eppendorf tubes.
  34. Add 700 μL of freshly prepared LiPEG buffer to each tube, invert three times, and rotate for 30 min at 30 °C.

**LiPEG Buffer**

**x 10 TE Solution**

**Material**

**Final Volume**

**Material**

**Final Volume**
50% PEG560 µL1 M Tris pH 85 mL1 M LiAc70 µL0.5 M EDTA1 mLx 10 TE70 µLDDWUp to 50 mL

35. Add 86 µL of DMSO, mix gently by flipping the tube, and heat shock at 42 °C for 10 min.
36. Centrifuge at 6–8000 rpm for 30 s and remove the supernatant. Resuspend the cells with 100 μL sterile water gently.

Note: Be gentle as possible at this step if high efficiency is important.

37. Plate the transformation mixes on 120 × 120 mm SD-Leu plates. Incubate the plates for 2–4 days to recover transformants.
38. Scrape all colonies from the plates and combine them in a 50 mL Falcon tube. Centrifuge at 3000× *g* and remove supernatant. Resuspend with 5 mL SD-leu, add 25% glycerol, and keep at −80.

### 4.7. FACS Isolation of Cells Having Different GFP/Cherry Ratios on BD FACSAria

Timing: 1 day

This part describes the method for sorting yeast cells into four populations based on their GFP to Cherry ratio. On the day of sorting, optimum sorting accuracy was attained by performing an automatic drop delay setup with BD Accudrop beads (BD Biosciences, Catalogue No. 345249) according to the manufacturer’s recommendation. Cytometer Setup &Tracking was performed before analysis using the CS&T beads (BD Biosciences, Catalogue No. 655050). A nozzle of 70 μm should be used while sorting.

39. Grow cells for 5–6 h and dilute to OD600 of ~0.002–0.005 in 10 mL SD-Leu.
40. Grow cells at 30 °C for about 14–18 h to reach 0.5–0.7 OD.
41. Keep cells at Room temperature for 30 min before sorting.
42. Add 0.01% sodium azide.
43. Run cherry-expressing and GFP-expressing cells to exclude cherry-negative and GFP-negative cells, respectively.
44. Gate single cells based on their forward and side scatter parameters.
45. Run the empty plasmid containing cells to optimize the population’s gating.
46. Divide the cells into four bins based on their GFP to mCherry ratio.
47. Collect 2 million of each population in 4 mL tubes that have 0.5 mL of SD-leu media.

### 4.8. Plasmid Extraction and Library Preparation for Sequencing

Timing: 2 days

In this step, plasmids were purified from each population and then used to generate amplicons suitable for Illumina NextSeq sequencing. Illumina adapters and indexation were added to the DNA library through two rounds of PCR.

48. Collect sorted cells by centrifugation at 10,000× *g* for 5 min. Carefully take out the supernatant by slow pipetting.
49. Immediately purify the plasmids by following the manufacturer’s instructions for the Zymoprep yeast Plasmid Miniprep II kit. Quantify the concentration by Nanodrop.
50. Prepare the first PCR as follows:

**Reagent**

**Amount**

**Final Concentration**
x 2 KAPA HiFi HotStart ReadyMix10 µLx 1P3-For0.75 µL300 nMP4-rev0.75 µL300 nMDNAx µL20 ngDDWx µLup to 25µL

51. Run the PCR:

**Steps**

**Temperature**

**Time**

**Cycles**
Initial Denaturation95 °C3 min1Denaturation98 °C20 s18 cyclesAnnealing60 °C15 s
Extension72 °C15 s
Final extension72 °C1 min
Hold4 °Cforever

52. Load 2 μL of the reaction on a 2% TAE/agarose gel and run at constant 100 V for 30 min.
53. Purify the PCR product using AMPure XP beads. Quantify the DNA using NanoDrop.
54. Perform and run the second PCR reaction with the Illumina primers to add the indexation. In the second PCR, use 50 ng of DNA templates and run the PCR program for 12 cycles.
55. Purify the PCR product using AMPure XP beads and quantify the DNA.
56. Pool the PCR products at a 1:1 ratio and load on 2% TAE/agarose gel. Excise the band of the library from the gel using a sterile scalpel and extract the DNA from the gel.
57. Quantify the concentration and send it for sequencing. We recommend a 4 nM final concentration of your sequencing library.

### 4.9. Peptide Alignment and Quantification 

An efficient data structure can be used to organize the sequencing data.

58. Trim nucleotide prefixes, including PCR barcodes, to yield DNA fragments consisting of shorter and more relevant sequences.
59. Use the Bowtie2 Python package for alignment, allowing a single mismatch, to yield a high coverage of the library.
60. Assess the appearance of each of the assigned peptides in the different FACS bins. Normalize the total read number of each bin to 1 × 10^6^ reads/bin.

### 4.10. Extraction of Protein Stability Index (PSI)

61. To calculate the contribution of each peptide to the stability of the fused GFP, determine its average position between the four bins, termed protein stability index (PSI) [17], using the equation: ${PSI}_{i}=\frac{\sum_{g\in [1,4]}g{\times f}_{i,g}}{\sum_{g\in [1,4]}f_{i,g}}$ where g is the gate number, ${\cdot f}_{i,g}$ is the frequency of the peptide i at the gate g. This yields a number between 1–4 that reflects peptide stability. The PSI of each 17 amino acid tile was assigned to the central amino acid (position nine; Figure 3b, black dots).
62. To obtain PSI for the peptide-devised proteome, average PSI values of all peptides harboring each amino acid (Typically, three tiles). Window-smooth this profile by a five-residue (plus/minus two) running median (Figure 3b, black line) [13,14].

### 4.11. Demonstration of the Use of a Training Model

A machine learning approach, using logistic regression, was employed to calculate the probability of any region in the proteome contributing to protein degradation and further disclose the unique characteristics of degron determinants.

Empirical PSI values served as the training dataset. Fragments with low read count (<50 counts at all FACS bins combined) were filtered out. The data was classified as stable or unstable, based on their PSI values. Any value smaller than 2.2 was considered unstable and was classified as “one” on a binary scale, while values greater than 2.8 were considered stable and classified as “zero”. Mid-range values (2.2 < PSI < 2.8) were omitted from the training set to reduce noise in the output model. To prevent overfitting behavior, ridge regularization with a value of λ = 0.001 was used for training.

The resulting product of the training model is a function that returns a probability score for a peptide to be a degron. As this function receives a peptide with a length of 17 amino acids and returns a value between zero and one, each amino acid residue within a protein (excluding the eight amino acids at the ends) can receive a prediction value. Together, these values can be fitted into a curve (using the cfit function in Matlab) to yield a continuous trending line of stability along the protein (see Figure 3b).

### 4.12. Demonstration of Data Analysis

We applied the QCDPred on the yeast proteome, which was retrieved from UniProt [20], comprising a total of 6617 protein sequences. Then, we used the peptide sequences and their degron probability to extract different features such as:

(1) Hydrophobicity

**Figure 4:**
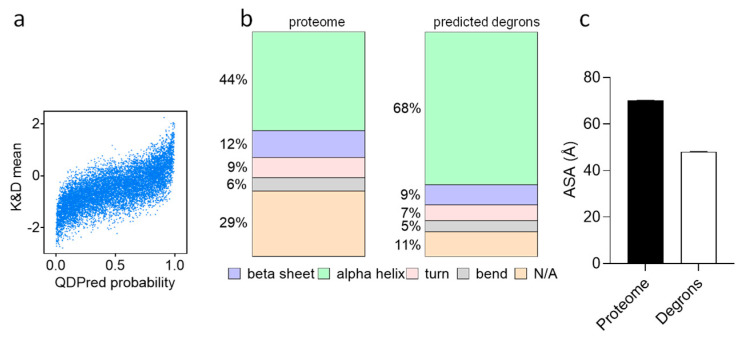
Properties of the yeast degronome. (**a**) A plot of Kyte–Doolittle hydrophobicity score of all peptides in the screen versus QCDPred probability. (**b**) Classification and relative proportions of protein secondary structure within the yeast proteome versus that of QCDPred-predicted degrons, based on the AlphaFold Protein Structure Database. (**c**) Accessible surface area (ASA) of amino acids within degrons compared to the entire proteome. Mann–Whitney U test shows a significance of *p* < 0.0001.

The correlation between degron probability and hydrophobicity of peptides was assessed using the Kyte–Doolittle scoring chart [21] (Figure 4a), however, any other hydrophobicity scale can be used. To do so, Kyte–Doolittle scores of each amino acid within each peptide were summed and averaged over the peptide length to obtain the peptide hydrophobicity score.

(2) Secondary Structure Analyses

To determine the secondary structure of peptides/degrons (helix, beta sheet, turn, bend, others), structural data needs to be collected. In our work, the AlphaFold 2.0 database was used to retrieve structural data for all proteins (out of complex) to yield more accurate results.

Using the AlphaFold-generated CIF structure file, the secondary structure can be determined for every amino acid in a protein and intersected with other properties such as degron prediction to obtain the secondary structure prevalence within the degron population.

(3) Accessible Surface Area (ASA)

Accessible Surface Area can be determined by utilizing the AlphaFold dataset and the FreeSASA 2.0 Python tool [22]. The algorithm assigns an ASA value to each amino acid of the yeast proteome and determines its accessibility region to the cytoplasm based on 3D atom distances, inferred from the related AlphaFold PDB files.

## 5. Results

The yGPS-P method was previously used for identifying QCAP degrons in the yeast *Saccharomyces Cerevisiae*. Using a synthetic library, fused to the C-terminus of GFP we isolated four plasmid populations with distinct GFP-to-Cherry ratios. Scoring each peptide, we next determined their PSI score. Using an advanced machine-learning algorithm, each amino acid in the yeast proteome was scored for its probability to be part of a degron. The new information was used for determining the amino acid and secondary structure preferences of the yeast degronome. We found that the QCDPred algorithm points to a preference for hydrophobic amino acids (Figure 4a). Cross-referencing the predicted yeast degronome with the AlphaFold yeast structure database [23,24], we detected enrichment of alpha-helices in QCAP degrons (Figure 4b). To evaluate the exposure and accessible surface area of amino acids within any protein, the AlphaFold structural PDB was used. We found that, in general, degrons are less solvent accessible, compared to the entire proteome (Figure 4c).

An important feature of the yeast QCAP ubiquitination machinery is that, unlike humans, it does not significantly encompass E3 ligases of the cullin RING family that recognize C-terminal amino acids as degrons. Consequently, C-terminal degrons were not enriched in the degron population of the yGPS-P screen [14]. Instead, QCAP degrons were found to be mostly internal and their QCDPred projected localization fit well with the published literature. The substantial enrichment of degrons in alpha helix secondary structure (Figure 4b), implies that a significant portion of the fused peptides can gain a secondary structure in the context of the GFP fusion. Thus, our data indicate that a C-terminally fused library of peptides that emerged from full-length proteins can be used to determine internal QCAP degrons.

Our observation that QCAP degrons are less susceptible to the solvent is not surprising; it is now well established that proteins tend to fold with their hydrophobic regions frequently buried at the protein core [25]. This raises the question of how QCAP degrons are being exposed during targeting of the UPS. Unlike an isolated environment, in which misfolded proteins can gain multiple extensively misfolded protein states [26], in vivo, proteins are likely to be in constant equilibrium between partially/mildly misfolded states, owing to barriers in their energy landscape. It is plausible that mild changes to the protein structure, mostly affecting its tertiary organization, are sufficient to expose well-folded hydrophobic helices that trigger the recruitment of E3 ligases. However, the underlying mechanisms and the contribution of protein amino acid sequences and structures to in vivo protein misfolding are yet to be determined.

Notably, yGPS-P can be utilized for other applications as well. For example, it can be used to identify stabilizing and/or destabilizing amino acids within a protein of interest, as previously conducted for the yeast Mat alpha2 protein [27]. Low-fidelity PCR or saturated mutagenesis can be used for preparing a large number of variants that can be sorted by FACS. Changes in PSI compared to the wild-type protein will indicate effects on stability. This is a very sensitive and robust way of determining the importance of specific amino acids to overall protein stability.

## Figures and Tables

**Table 1:** Bacteria and yeast strains.

Strains	Genotype	Source
10-Beta electrocompetent bacteria	*Δ(ara-leu) 7697 araD139 fhuA ΔlacX74 galK16 galE15 e14- Φ80dlacZΔM15 recA1 relA1 endA1 nupG rpsL (StrR) rph spoT1 Δ(mrr-hsdRMS-mcrBC)*	New England Biolabs Cat# C3020K
TRy1392 yeast cells	*MATa met15∆0, ura3∆0, leu2∆0, his3∆1, pdr5::KanMX*	[19]

**Table 2:** Primers used.

Primer	Sequence
P1-F	GATCAGCTGGCTCACCCG
P2-R	CTAATTACATGATCAGTCAGCTAGC
P3-F	TCGTCGGCAGCGTCAGATGTGTATAAGAGACANNNGGATCAGCTGGCTCACCC
P4-R	GTCTCGTGGGCTCGGAGATGTGTATAAGAGACAGANNNACATAACTAATTACATGATCAGTCAGCT

**Table 3:** Commercial kits used.

Kit	Source	Cat#
NucleoSpin Gel and PCR Clean-up kit	MACHEREY-NAGEL	740609
KAPA HiFi HotStart ReadyMix PCR Kit	Roche	KK2602
PureLink^®^ HiPure plasmid filter Midiprep kit	Invitrogen	K210014
Zymoprep yeast Plasmid Miniprep II	Zymo Research	D2004
Gibson Assembly Master Mix	New England Biolabs	M5510
Phusion High-Fidelity DNA Polymerase	New England Biolabs	M0530S

**Table 4:** Chemicals used.

Chemical	Source	Cat#
dNTP set, 100 mM solutions	New England Biolabs	N0442
Polyethylene Glycol 4000	Sigma-Aldrich	8.07490
Dithiothreitol	Formedium	3483-12-3
Tryptone	Thermo Fisher Scientific	211705
Peptone	Thermo Fisher Scientific	211677
Yeast Extract	Thermo Fisher Scientific	212750
Sodium chloride	Bio-Lab Chemicals	001903059400
Ampicillin sodium salt	Sigma	A0166
Bacto^TM^ dehydrated Agar	BD	214010
Gel Loading Dye, Purple (6X), no SDS	New England Biolabs	B7025
*Nhe*I restriction enzyme	New England Biolabs	R3131
*Xma*I restriction enzyme	New England Biolabs	R0180
Ethidium Bromide	Genesee Scientific	20276
1 kb DNA Ladder	New England Biolabs	N3232S
100 bp DNA Ladder	New England Biolabs	N3231S
Bacto agar	BD	214010
Glycerol	JT Baker	2136-01
Ethidium Bromide	Genesee Scientific	20276
1 kb DNA Ladder	New England Biolabs	N3232S
100 bp DNA Ladder	New England Biolabs	N3231S
100 bp DNA Ladder	New England Biolabs	N3231S
Bacto agar	BD	214010
Glycerol	JT Baker	2136-01
SeaKem^®^ LE Agarose	Lonza Bioscience	50002
D-Sorbitol	Sigma	S1876
Deoxyribonucleic acid sodium salt	Sigma	D1626
DMSO	Fluka	41650
EDTA	JT Baker	8993-01
Trizma base	Sigma	T1503
Sodium Azide	Sigma-Aldrich	S2002
AMPure XP beads	Beckman Coulter	A63880
Glucose	Carl Roth	X997.2
Lithium acetate	Sigma-Aldrich	L6883
Ammonium Sulfate	Sigma-Aldrich	A5132

**Table 5:** Equipment.

Key Equipment
Centrifuge
30 °C incubator
37 °C incubator
ECM 399 electroporator system
Nanodrop
BD FACS Aria sorter with 70 μm nozzle
Thermocycler
Scrapers

## Data Availability

Not applicable.
